# Supplementary material for: Ascent and Attachment in Pea Plants: A Matter of Iteration
Source: Plants (Basel). Author manuscript; Available in PMC 2024 May 30. (PMC11124904; doi:10.3390/plants13101389)
Supplement: Supplementary material [file EMS196358-supplement-Supplementary_material.pdf]

Supplementary information

Ascent and Attachment in Pea Plants: A Matter of Iteration

Silvia Guerra *, Giovanni Bruno, Andrea Spoto, Anna Panzeri, Qiuran Wang, Bianca Bonato, Valentina Simonetti and Umberto Castiello

Department of General Psychology, University of Padova, 35131 Padova, Italy
* Correspondence: silvia.guerra@unipd.it; Tel.: +39-049-827-1216

The file contains:

- Supplementary Figures S1 – S3
- Supplementary Tables S1 – S5

Table S1A. Mean and standard deviation for the average velocity and acceleration profile of circumnutation, distance from the origin of the plant to the center of circumnutation, and area of circumnutation concerning Experimental condition ('Support,' 'No Support') and Experimental Phase ('PRE,' 'POST').

	Velocity	Acceleration	Distance	Area
**Condition: Support**				
- PRE	.296 (.258)	.0023 (.0040)	8.43 (7.52)	26.4 (53.3)
- POST	.753 (.659)	.0059 (.0102)	18.8 (15.2)	210 (457)
**Condition: No Support**				
- PRE	.210 (.160)	.0023 (.0028)	5.21 (3.42)	7.58 (17.3)
- POST	.418 (.387)	.0036 (.0055)	10.7 (7.00)	55.9 (131)

Table S1B. Results from the lmer fitted models (Type III Wald chi-square tests) investigating the interaction between Experimental Condition ('Support,' 'No Support') and Experimental Phase ('PRE,' 'POST') for the four kinematical variables considered (scaled). Plant and Experimental Conditions were set as random intercept and random slope variables.

	$\chi 2$	df	Pr(>χ2)	R^2
**Velocity ~**				
(Intercept)	9.688	1	.001**	
Condition	8.614	1	.003**	
Phase	730.214	1	<.001***	
Phase*Condition	52.055	1	<.001***	
- Marginal R^2				.186
- Conditional R^2				.479
**Acceleration ~**				
(Intercept)	2.804	1	.093°	
Condition	.008	1	.099°	
Phase	109.252	1	<.001***	
Phase*Condition	9.390	1	.002**	
- Marginal R^2				.038
- Conditional R^2				.181
**Distance ~**				
(Intercept)	18.725	1	<.001***	
Condition	20.449	1	<.001***	
Phase	478.877	1	<.001***	
Phase*Condition	35.519	1	<.001***	
- Marginal R^2				.175
- Conditional R^2				.325
**Area ~**				
(Intercept)	11.842	1	<.001***	
Condition	17.896	1	<.001***	
Phase	235.725	1	<.001***	
Phase*Condition	42.041	1	<.001***	
- Marginal R^2				.089
- Conditional R^2				.201

Note. χ^2 = Chi-squared test; R^2 = Coefficient of determination; df = Degrees of Freedom. N observations = 3117. ° = $p < .100$; * = $p < .050$; ** = $p < .010$; *** = $p < .001$.

Table S1C. Post-hoc analysis ("emmeans" contrast) on the significant interaction effects (Experimental Condition*Experimental Phase) detected in the previous four models.

	estimate	SE	df	Z ratio	*p*-value
**Velocity ~**					
Sup PRE – Sup POST	-.946	.206	Inf	-27.022	<.001
No PRE – No POST	-.524	.049	Inf	-11.185	<.001
Sup PRE – No PRE	.184	.209	Inf	.881	.814
Sup POST – No POST	.606	.206	Inf	2.935	.017
**Acceleration ~**					
Sup PRE – Sup POST	-.450	.142	Inf	-10.452	<.001
No PRE – No POST	-.229	.043	Inf	-3.991	<.001
Sup PRE – No PRE	.014	.057	Inf	.093	.999
Sup POST – No POST	.233	.142	Inf	1.646	.353
**Distance ~**					
Sup PRE – Sup POST	-.862	.039	Inf	-21.883	<.001
No PRE – No POST	-.470	.052	Inf	-8.920	<.001
Sup PRE – No PRE	.259	.149	Inf	1.738	.304
Sup POST – No POST	.651	.144	Inf	4.522	.<.001
**Area ~**				.	
Sup PRE – Sup POST	-.665	.043	Inf	-4.230	<.001
No PRE – No POST	-.197	.058	Inf	-3.417	.003
Sup PRE – No PRE	.044	.128	Inf	.346	.986
Sup POST – No POST	-512	.121	Inf	4.230	<.001

Note. Sup PRE = Support, PRE phase; Sup POST = Support, POST phase; No PRE = No Support, PRE phase; No POST = No Support, POST phase. SE = Standard Error, df = Degrees of Freedom.

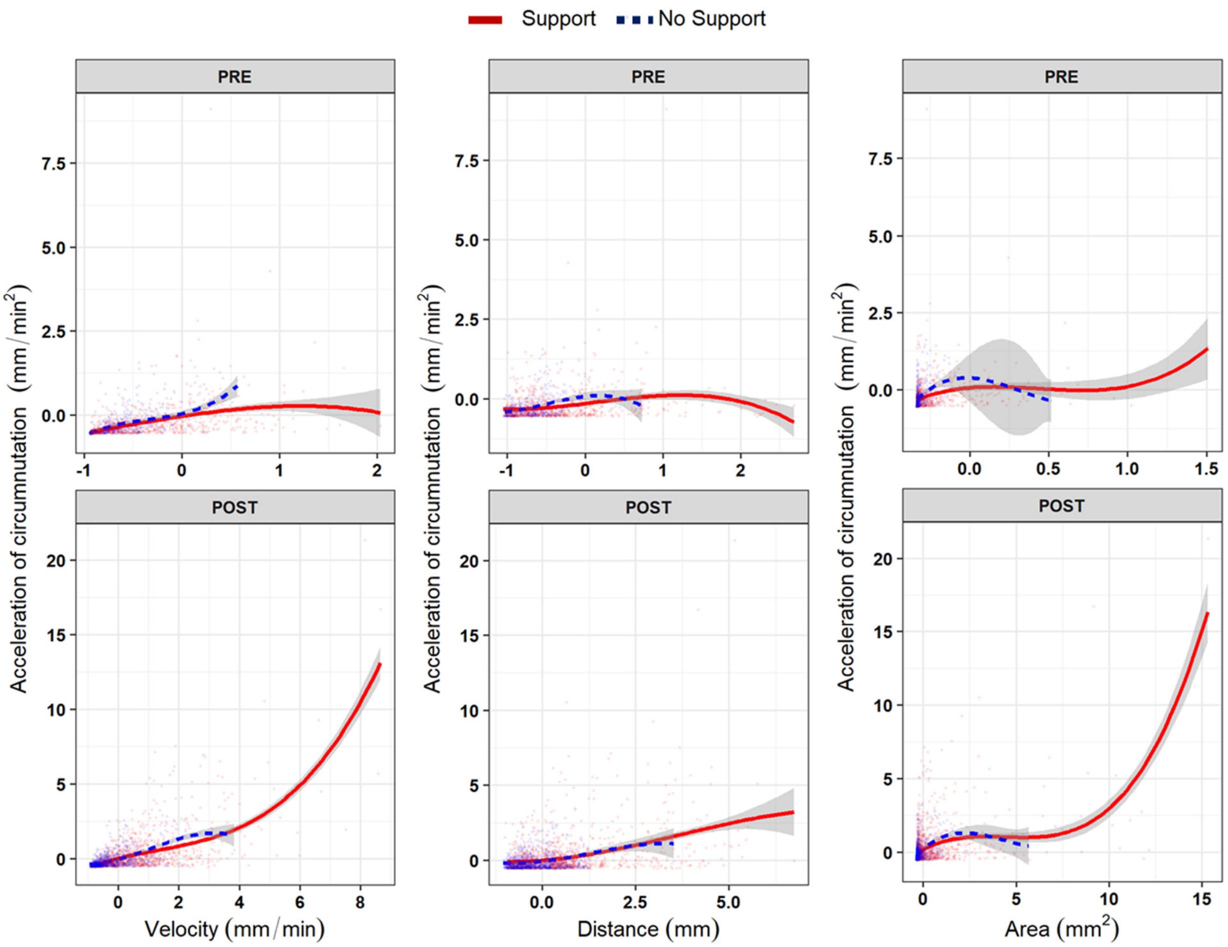

Figure S1A. B-spline curves (degrees of freedom = 3) for the non-linear relationship across Experimental Phases (row facets) between the acceleration of circumnutation (as the scaled dependent variable, y axes) and the other three kinematic variables (column facets): the average velocity of circumnutation, distance from the origin of the plant to the center of circumnutation and area of circumnutation. Data represent the sole activity of the apex of the plant. The red solid line represents the 'Support' condition, and the blue dashed line represents the 'No Support' condition.

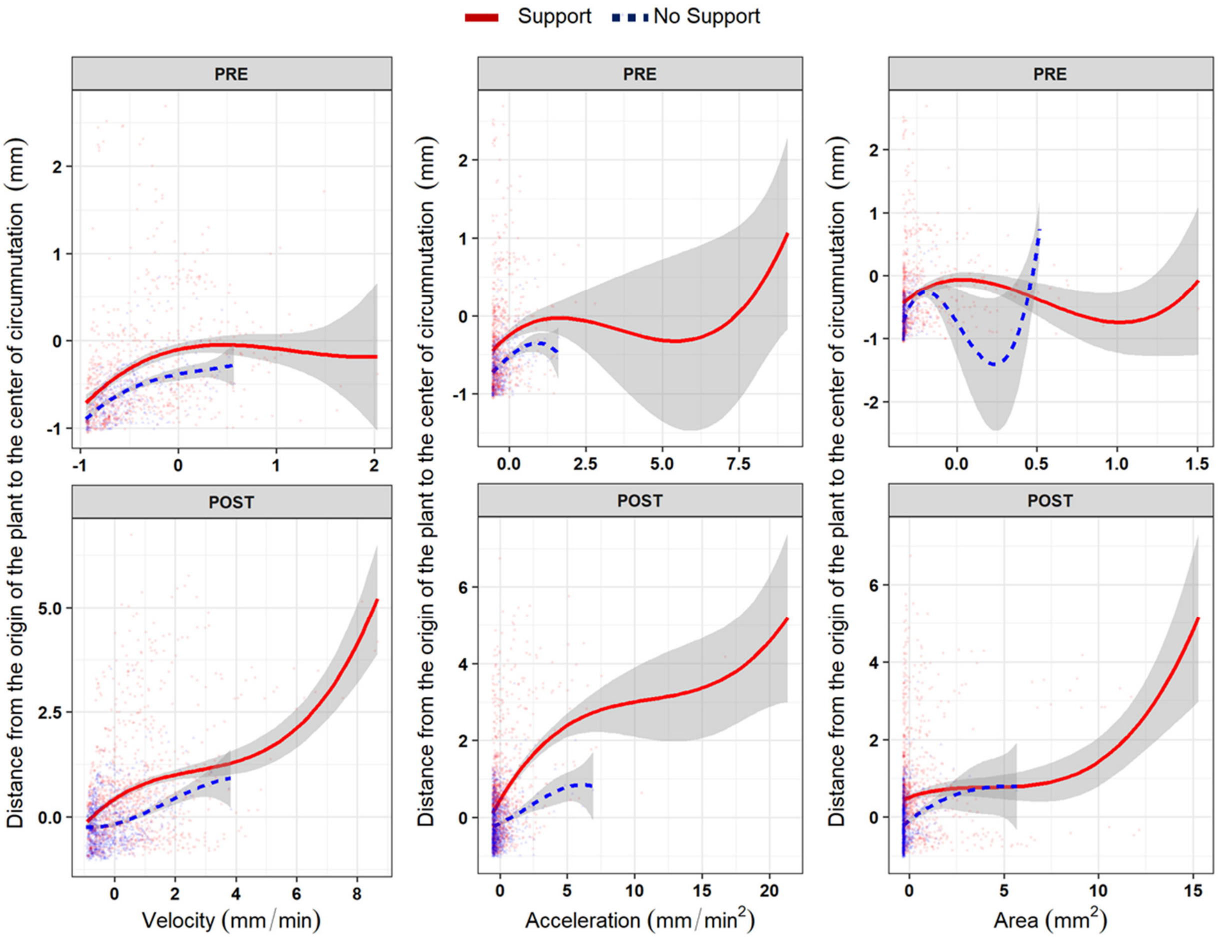

Figure S1B. B-spline curves (degrees of freedom = 3) for the non-linear relationship across Experimental Phases (row facets) between the distance from the origin of the plant to the center of circumnutation (as the scaled dependent variable, y axes) and the other three kinematic variables (column facets): the average velocity of circumnutation, acceleration of circumnutation and area of circumnutation. Data represent the sole activity of the apex of the plant. The red solid line represents the 'Support' condition, and the blue dashed line represents the 'No Support' condition.

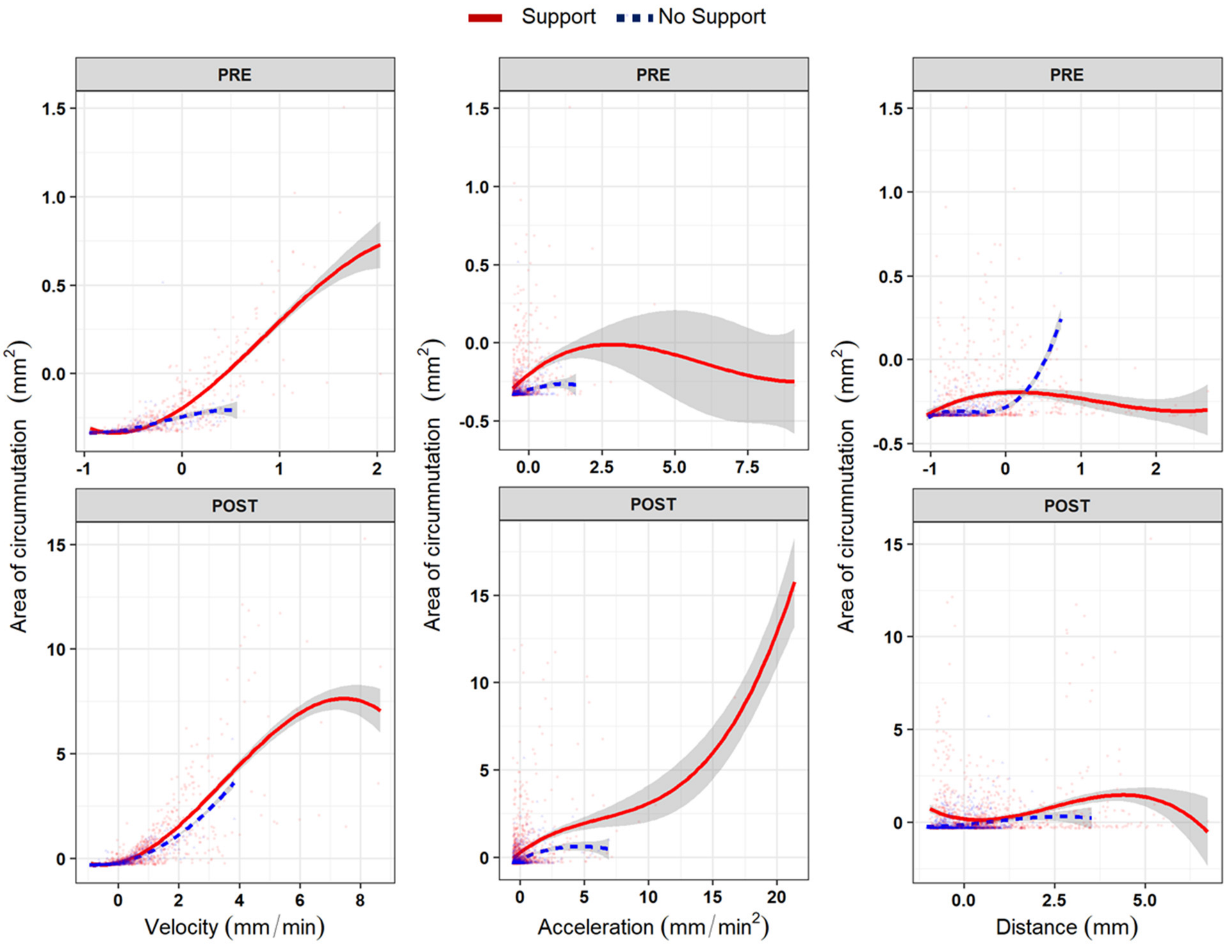

Figure S1C. B-spline curves (degrees of freedom = 3) for the non-linear relationship across Experimental Phases (row facets) between an area of circumnutation (as the scaled dependent variable, y axes) and the other three kinematic variables (column facets): the average velocity of circumnutation, acceleration of circumnutation and distance from the origin of the plant to the center of the circumnutation. Data represent the sole activity of the apex of the plant. The red solid line defines the 'Support' condition, and the blue dashed line represents the 'No Support' condition.

Table S2A. Results from the generative additive model setting the average velocity of circumnutation as the dependent variable (scaled) and the other three kinematic variables as smoothed terms for the interaction between Experimental Condition ('Support,' 'No Support') and Experimental Phase ('PRE,' 'POST'). The plant was set as a random smoothed intercept.

Parametric coefficients	**Estimate**	**SE**	**t value**	**Pr(>|t|)**
(Intercept)	0.506	.025	20.21	<.001***
Approximate significance of smooth terms	**edf**	**Ref.df**	**F value**	***p*-value**
s(Area): Stim PRE	4.701	4.919	227.758	<.001***
s(Area): Stim POST	8.187	8.679	633.155	<.001***
s(Area): No PRE	2.952	2.992	36.342	<.001***
s(Area): No POST	6.001	6.304	291.224	<.001***
s(Distance): Stim PRE	2.648	3.282	3.273	.021*
s(Distance): Stim POST	1.002	1.003	219.425	<.001***
s(Distance): No PRE	2.718	3.114	1.380	.368
s(Distance): No POST	3.682	4.490	8.053	<.001***
s(Acceleration): Stim PRE	3.655	4.361	11.304	<.001***
s(Acceleration): Stim POST	7.961	8.625	68.015	<.001***
s(Acceleration): No PRE	1.000	1.001	20.574	<.001***
s(Acceleration): No POST	2.162	2.681	31.639	<.001***
s (ID Plant) as random effect	22.045	23.00	43.919	<.001***
Adjusted R^2 = .898				

Note. SE = Standard Error, edf = effective degrees of freedom (as an index of smoothness complexity); Ref.df = reference degrees of freedom. Sup PRE = Support, PRE phase; Sup POST = Support, POST phase; No PRE = No Support, PRE phase; No POST = No Support, POST phase. N observations = 3115. ° = $p < .100$; * = $p < .050$; ** = $p < .010$; *** = $p < .001$.

Table S2B. Results from the generative additive model setting acceleration of circumnutation as dependent variable (scaled) and the other three kinematic variables as smoothed terms, controlling for the interaction between Experimental Condition ('Support,' 'No Support') and Experimental Phase ('PRE,' 'POST'). The plant was set as a random smoothed intercept.

Parametric coefficients	**Estimate**	**SE**	**t value**	**Pr(>|t|)**
(Intercept)	.004	.001	18.8	<.001***

Approximate significance of smooth terms	**edf**	**Ref.df**	**F value**	**p-value**
s(Area): Stim PRE	2.570	3.029	3.524	.014*
s(Area): Stim POST	8.622	8.952	43.971	<.001***
s(Area): No PRE	1.002	1.003	4.200	.040*
s(Area): No POST	1.939	2.415	14.509	<.001***
s(Distance): Stim PRE	1.007	1.014	0.323	.571
s(Distance): Stim POST	2.112	2.653	15.463	<.001***
s(Distance): No PRE	1.004	1.007	0.362	.550
s(Distance): No POST	2.590	3.237	1.398	.230
s(Speed): Stim PRE	1.460	1.784	28.687	<.001***
s(Speed): Stim POST	7.726	8.560	73.285	<.001***
s(Speed): No PRE	1.003	1.006	24.233	<.001***
s(Speed): No POST	5.200	6.086	26.770	<.001***
s (ID Plant) as random effect	14.761	23.000	1.906	<.001***
Adjusted R^2 = .542				

Note. SE = Standard Error, edf = effective degrees of freedom (as an index of smoothness complexity); Ref.df = reference degrees of freedom. Sup PRE = Support & PRE phase; Sup POST = Support & POST phase; No PRE = No Support & PRE phase; No POST = No Support & POST phase. N observations = 3115. ° = $p < .100$; * = $p < .050$; ** = $p < .010$; *** = $p < .001$.

Table S2C. Results from the generative additive model setting distance from the origin of the plant to the center of circumnutation as dependent variable (scaled) and the other three kinematic variables as smoothed terms, controlling for the interaction between Experimental Condition ('Support,' 'No Support') and Experimental Phase ('PRE,' 'POST'). The plant was set as a random smoothed intercept.

Parametric coefficients	**Estimate**	**SE**	**t value**	**Pr(>|t|)**
(Intercept)	103.39	12.15	8.51	<.001***

Approximate significance of smooth terms	**edf**	**Ref.df**	**F value**	***p*-value**
s(Speed): Stim PRE	3.699	4.486	28.116	<.001***
s(Speed): Stim POST	8.846	8.991	638.615	<.001***
s(Speed): No PRE	1.008	1.013	1.131	.289
s(Speed): No POST	4.526	5.430	83.980	<.001***
s(Distance): Stim PRE	1.003	1.006	22.027	<.001***
s(Distance): Stim POST	8.152	8.781	16.965	<.001***
s(Distance): No PRE	2.349	2.848	4.914	.003**
s(Distance): No POST	1.011	1.022	1.185	.279
s(Acceleration): Stim PRE	1.497	1.818	0.377	.611
s(Acceleration): Stim POST	8.680	8.966	85.308	<.001***
s(Acceleration): No PRE	1.006	1.011	0.093	.769
s(Acceleration): No POST	1.880	2.339	3.783	.018*
s (ID Plant) as random effect	21.728	23.000	17.321	<.001***

Adjusted R^2 = .787

Note. SE = Standard Error, edf = effective degrees of freedom (as an index of smoothness complexity); Ref.df = reference degrees of freedom. Sup PRE = Support & PRE phase; Sup POST = Support & POST phase; No PRE = No Support & PRE phase; No POST = No Support & POST phase. N observations = 3115. ° = p < .100; * = p < .050; ** = p < .010; *** = p < .001.

Table S2D. Results from the generative additive model setting area of circumnutation as dependent variable (scaled) and the other three kinematic variables as smoothed terms, controlling for the interaction between Experimental Condition ('Support,' 'No Support') and Experimental Phase ('PRE,' 'POST'). The plant was set as a random smoothed intercept.

Parametric coefficients	**Estimate**	**SE**	**t value**	**Pr(>|t|)**
(Intercept)	14.033	1.051	13.35	<.001***
Approximate significance of smooth terms	**edf**	**Ref.df**	**F value**	***p*-value**
s(Speed): Stim PRE	3.179	3.864	24.508	<.001***
s(Speed): Stim POST	6.825	7.917	34.495	<.001***
s(Speed): No PRE	2.334	2.906	1.043	.280
s(Speed): No POST	1.776	2.252	11.487	<.001***
s(Area): Stim PRE	3.294	3.702	9.130	<.001***
s(Area): Stim POST	8.149	8.715	14.546	<.001***
s(Area): No PRE	1.000	1.001	6.307	.012*
s(Area): No POST	1.684	2.071	1.977	.135
s(Acceleration): Stim PRE	1.010	1.019	0.057	.822
s(Acceleration): Stim POST	5.680	6.731	8.425	<.001***
s(Acceleration): No PRE	1.002	1.004	0.074	.789
s(Acceleration): No POST	1.004	1.008	2.152	.141
s (ID Plant) as random effect	22.381	23.000	33.278	<.001***
Adjusted R^2 = .468				

Note. SE = Standard Error, edf = effective degrees of freedom (as an index of smoothness complexity); Ref.df = reference degrees of freedom. Sup PRE = Support & PRE phase; Sup POST = Support &POST phase; No PRE = No Support & PRE phase; No POST = No Support & POST phase. N observations = 3115. ° = $p < .100$; * = $p < .050$; ** = $p < .010$; *** = $p < .001$.

Table S3A. Results from the lmer fitted models (Type III Wald chi-square tests) investigating the three-parties interaction between Experimental Condition ('Support,' 'No Support') and anatomical landmark of the plant ('Apex,' 'Tendril') throughout Leaf ('Third last,' 'Second last,' 'Last') for the four kinematical variables considered (scaled). The plant was set as a random intercept for each model.

	**$\chi 2$**	**df**	**Pr(>$\chi 2$)**	**R^2**
**Velocity ~**				
(Intercept)	4.32	1	.037**	
Condition: Point: Leaf	4017.53	11	<.001***	
- Marginal R^2				
- Conditional R^2				.323
				.588
**Acceleration ~**				
(Intercept)	7.12	1	.007*	
Condition: Point: Leaf	895.68	11	<.001***	
- Marginal R^2				.125
- Conditional R^2				.260
**Distance ~**				
(Intercept)	16.84	1	<.001***	
Condition: Point: Leaf	1207.36	11	<.001***	
- Marginal R^2				.166
- Conditional R^2				.320
**Area ~**				
(Intercept)	.051	1	.821	
Condition: Point: Leaf	1302.22	11	<.001***	
- Marginal R^2				.164
- Conditional R^2				.406

Note. χ^2 = Chi-squared test; R^2= Coefficient of determination; df = Degrees of Freedom. ° = $p < .100$; * = $p < .050$; ** = $p < .010$; *** = $p < .001$.

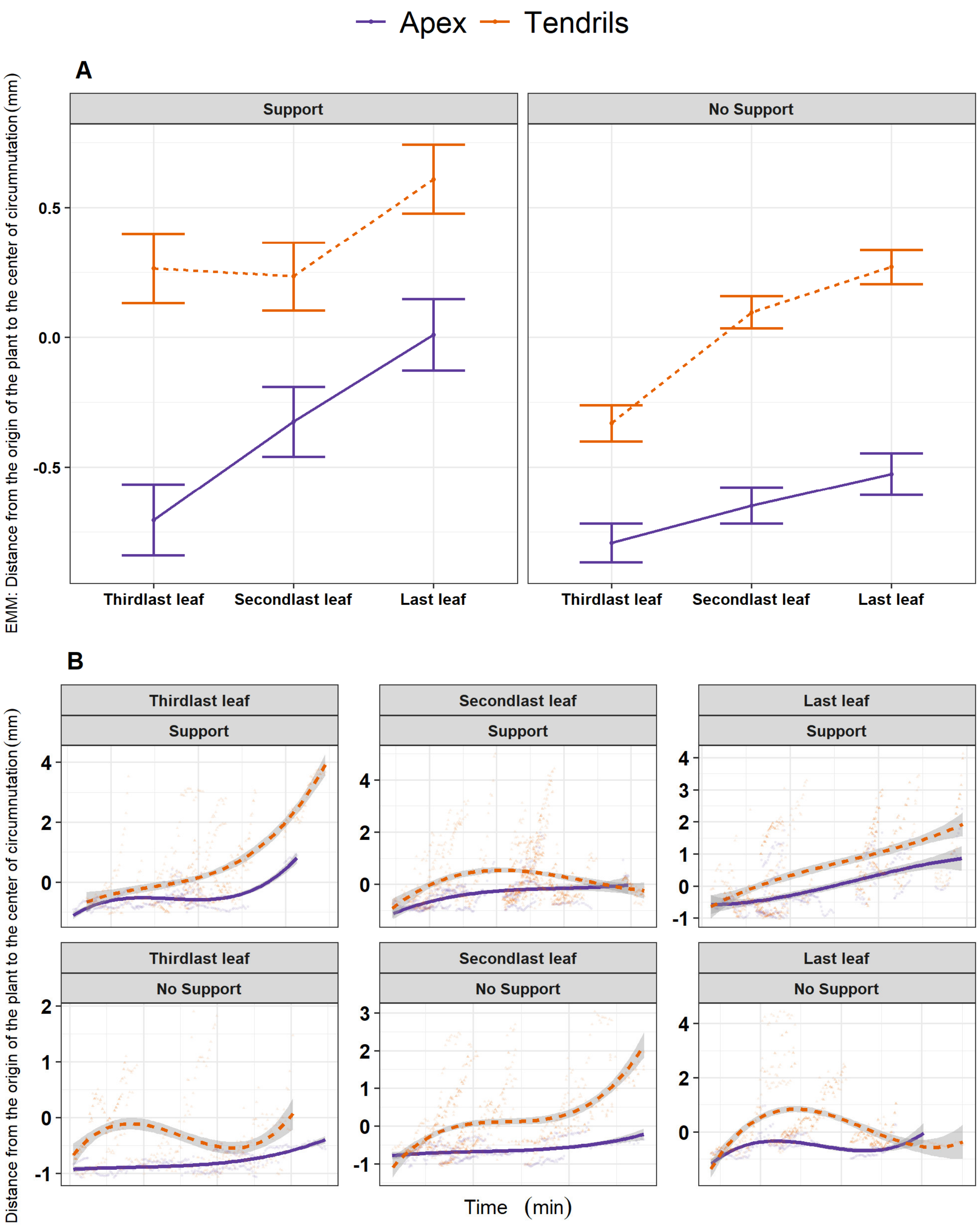

Figure S2A. (A) Graphical representation of post-hoc analysis for the interaction between Experimental Condition ('Stimulus,' 'No Stimulus'), anatomical landmark of the plant ('Apex,' 'Tendrils') and Leaf ('Third last,' 'Second last,' 'Last') for the estimation of the distance from the origin of the plant to the center of circumnutation (scaled) in the 'Third last,' 'Second last' and 'Last' leaf. **(B)** For descriptive purposes, the distribution of the same kinematical variable is represented as smoothed across the three last leaves of interest, controlling for the same experimental factors. Tendrils are represented with the orange-dashed line and the apex with the violet-solid line.

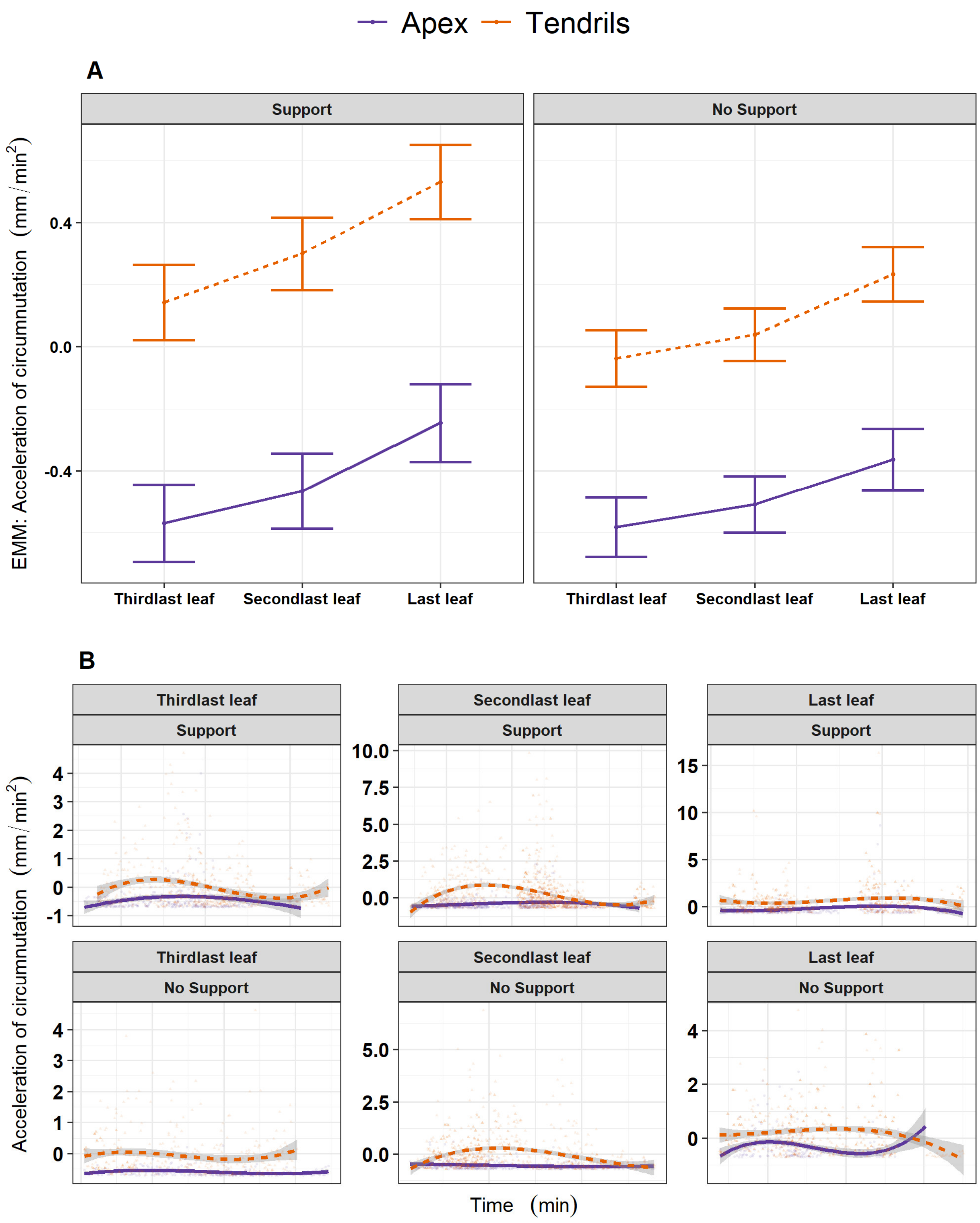

Figure S2B. (A) Graphical representation of post-hoc analysis for the interaction between Experimental Condition ('Stimulus,' 'No Stimulus'), anatomical landmark of the plant ('Apex,' 'Tendrils') and Leaf ('Third last,' 'Second last,' 'Last') for the estimation of the acceleration of circumnutation (scaled). **(B)** For descriptive purposes, the distribution of the same kinematical variable is represented as smoothed across the three last leaves of interest, controlling for the same experimental factors. Tendrils are represented with the orange-dashed line. The apex is represented with the violed-solid line.

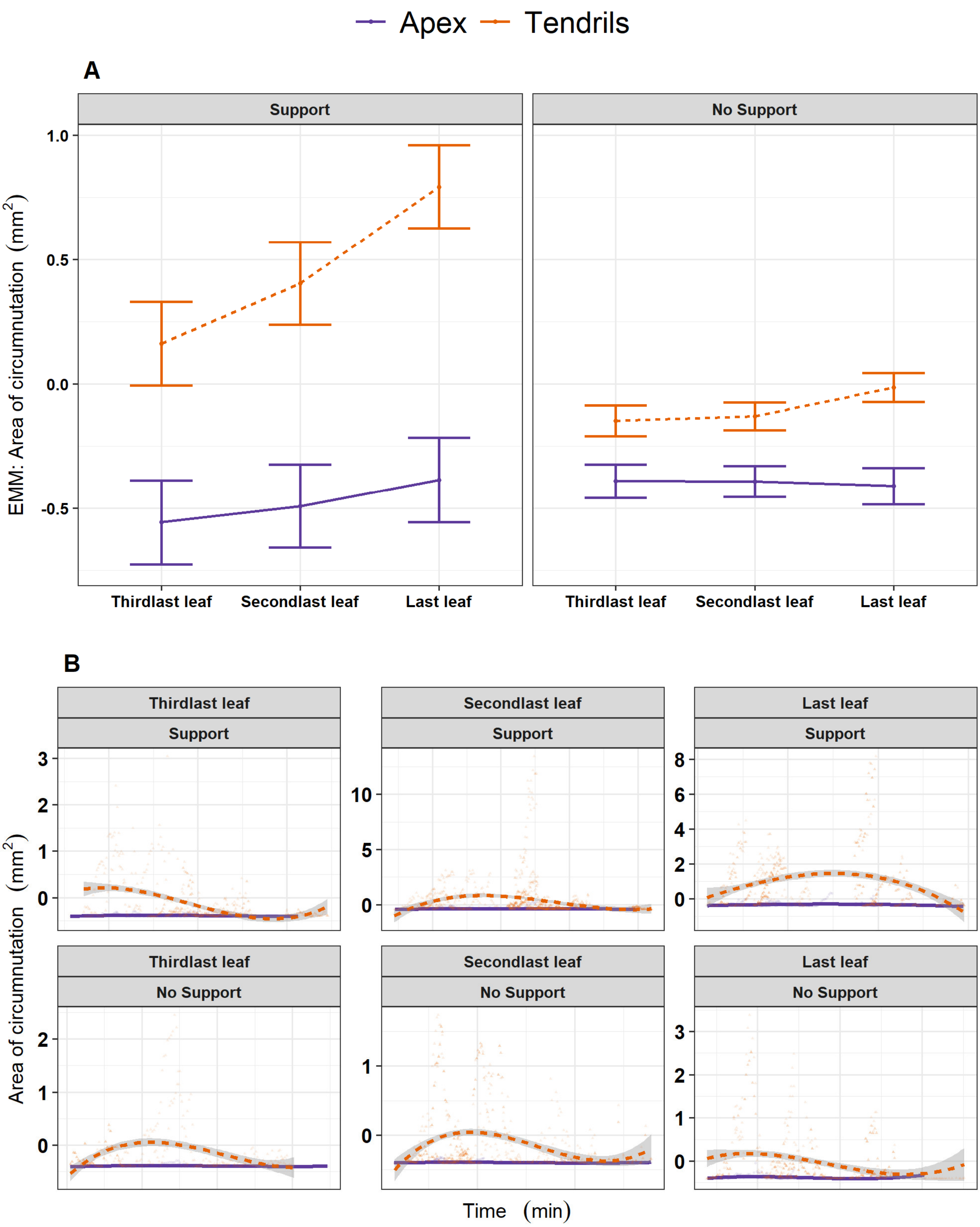

Figure S2C. (A) Graphical representation of post-hoc analysis for the interaction between Experimental Condition ('Stimulus,' 'No Stimulus'), anatomical landmark of the plant ('Apex,' 'Tendrils') and Leaf ('Third last,' 'Second last,' 'Last') for the estimation of the area of circumnutation (scaled). **(B)** For descriptive purposes, the distribution of the same kinematical variable is represented as smoothed across the three last leaves of interest, controlling for the same experimental factors. Tendrils are represented with the orange-dashed line and the apex with the violed-solid line.

Table S3B. Post-hoc analysis ("emmeans" contrast) for the three-parties interaction effects of the models described in Table S3-A.

	contrast							est	se	df	z ratio	*p*-value
**Velocity ~**												
	Sup	Apex	3L	-	Sup	Apex	2L	-.093	050	Inf	-1.884	769
	Sup	Apex	2L	-	Sup	Apex	1L	-.228	.050	Inf	-4.581	<.001
	Sup	Tend	3L	-	Sup	Tend	2L	-.255	.036	Inf	-7.090	<.001
	Sup	Tend	2L	-	Sup	Tend	1L	-.438	.034	Inf	-12.668	<.001
	No	Apex	3L	-	No	Apex	2L	-.036	.054	Inf	-.664	1.00
	No	Apex	2L	-	No	Apex	1L	-.071	.059	Inf	-1.206	.989
	No	Tend	3L	-	No	Tend	2L	-.107	.044	Inf	-2.446	.374
	No	Tend	2L	-	No	Tend	1L	-.220	.040	Inf	-5.458	<.001
**Acceleration ~**	**contrast**							**est**	**se**	**df**	**z ratio**	***p*-value**
	Sup	Apex	3L	-	Sup	Apex	2L	-0.104	0.065	Inf	-1.592	0.912
	Sup	Apex	2L	-	Sup	Apex	1L	-0.220	0.066	Inf	-3.349	0.039
	Sup	Tend	3L	-	Sup	Tend	2L	-0.157	0.048	Inf	-3.302	0.045
	Sup	Tend	2L	-	Sup	Tend	1L	-0.231	0.046	Inf	-5.08	<.001
	No	Apex	3L	-	No	Apex	2L	-0.074	0.071	Inf	-1.035	0.997
	No	Apex	2L	-	No	Apex	1L	-0.144	0.078	Inf	-1.853	0.788
	No	Tend	3L	-	No	Tend	2L	-0.076	0.058	Inf	-1.323	0.976
	No	Tend	2L	-	No	Tend	1L	-0.195	0.053	Inf	-3.684	0.012
**Distance ~**	**contrast**							**est**	**se**	**df**	**z ratio**	***p*-value**
	Sup	Apex	3L	-	Sup	Apex	2L	-0.378	0.063	Inf	-6.003	<.001
	Sup	Apex	2L	-	Sup	Apex	1L	-0.335	0.063	Inf	-5.318	<.001
	Sup	Tend	3L	-	Sup	Tend	2L	0.031	0.046	Inf	0.679	1.000
	Sup	Tend	2L	-	Sup	Tend	1L	-0.374	0.044	Inf	-8.552	<.001
	No	Apex	3L	-	No	Apex	2L	-0.144	0.068	Inf	-2.114	0.613
	No	Apex	2L	-	No	Apex	1L	-0.121	0.075	Inf	-1.618	0.903
	No	Tend	3L	-	No	Tend	2L	-0.426	0.055	Inf	-7.716	<.001
	No	Tend	2L	-	No	Tend	1L	-0.174	0.051	Inf	-3.432	0.030
**Area ~**	**contrast**							**est**	**se**	**df**	**z ratio**	***p*-value**
	Sup	Apex	3L	-	Sup	Apex	2L	-0.066	0.059	Inf	-1.12	0.994
	Sup	Apex	2L	-	Sup	Apex	1L	-0.105	0.059	Inf	-1.78	0.829
	Sup	Tend	3L	-	Sup	Tend	2L	-0.241	0.043	Inf	-5.652	<.001
	Sup	Tend	2L	-	Sup	Tend	1L	-0.388	0.041	Inf	-9.48	<.001
	No	Apex	3L	-	No	Apex	2L	0.002	0.064	Inf	0.03	1.000
	No	Apex	2L	-	No	Apex	1L	0.018	0.070	Inf	0.263	1.000
	No	Tend	3L	-	No	Tend	2L	-0.017	0.052	Inf	-0.336	1.000
	No	Tend	2L	-	No	Tend	1L	-0.117	0.047	Inf	-2.468	0.360

Note. Sup = Support, No = No Support; Apex = Apex; Tend = Tendril; 3L = Third last Leaf; 2L = Second last Leaf; 1L = Last Leaf. se = Standard Error, df = Degrees of Freedom.

Table S4A. Mean, standard deviation, and range (min, max) for the total number of circumnutations and switches across the three last leaves developed concerning Experimental Condition.

	Mean (SD)	Min	Max
**Circumnutations (Support)**			
- Third last leaf	33 (10.6)	10	51
- Second last leaf	34.1 (10.7)	6	61
- Last leaf	19.2 (11.6)	5	64
**Circumnutations (No Support)**			
- Third last leaf	42.8 (12.1)	11	70
- Second last leaf	48.1 (12.6)	23	67
- Last leaf	34.8 (14.1)	1	59
**Switches (Support)**			
- Third last leaf	4.40 (2.90)	0	9
- Second last leaf	2.79 (1.99)	0	7
- Last leaf	1.38 (1.30)	0	4
**Switches (No support)**			
- Third last leaf	5.37 (4.05)	0	16
- Second last leaf	6.12 (6.12)	1	11
- Last leaf	4.31 (2.85)	0	9

Table S4B. Results from the lmer fitted models (Type III Wald chi-square tests) investigating the interaction between Experimental Condition ('Support,' 'No Support') and Leaf ('Third last,' 'Second last,' 'Last') for the number of switch direction of circumnutations ('Clockwise' and 'Counterclockwise'). The plant was set as a random intercept for the two models.

	$\chi 2$	df	Pr(>$\chi 2$)	R^2
**Circumnutations ~**				
(Intercept)	275.958	1	<.001***	
Condition	3.587	2	.058°	
Leaf	191.105	2	<.001***	
Condition*Leaf	11.335	2	.003**	
- Marginal R^2				.373
- Conditional R^2				.609
**Switches ~**				
(Intercept)	172.130	1	< .001***	
Condition	1.569	1	.210	
Leaf	32.173	2	< .001***	
Direction	0.001	1	.970	
Condition*Leaf	13.225	2	.001**	
Condition*Direction	0.058	1	.809	
Leaf *Direction	0.664	2	.717	
Condition*Leaf *Direction	0.340	2	.844	
- Marginal delta R^2				.451
- Conditional delta R^2				.513

Note. χ^2 = chi-squared test; R^2= Coefficient of determination; df: degree of freedom; ° = $p < .100$; * = $p < .050$; ** = $p < .010$; *** = $p < .001$.

Table S4C. Post-hoc analysis ("emmeans" contrast) for the significant interaction effects (Experimental Condition*Leaf) detected on the two models described in Table S4-A and Table S4-B.

	estimate	SE	df	Z ratio	*p*-value
**Circumnutations ~**					
Sup 3L – Sup 2L	1.589	1.45	455.4	1.098	.882
Sup 2L – Sup 1L	15.727	1.270	452.7	7.173	<.001
Sup 3L – Sup 1L	17.316	1.53	459.5	11.310	<.001
No 3L – No 2L	8.689	3.65	30.5	2.378	.195
No 2L – No 1L	28.349	3.54	27.0	8.005	<.001
No 3L – No 1L	8.983	1.97	451.7	4.563	<.001
Sup 3L – No 3L	-7.100	3.75	33.7	-1.894	.423
Sup 2L – No 2L	-12.622	3.53	26.6	-3.577	.015
Sup 1L – No 1L	-15.433	3.61	29.1	-4.277	.002
**Switches ~**					
Sup 3L – Sup 2L	.450	.080	Inf	5.647	<.001
Sup 2L – Sup 1L	.682	.098	Inf	6.943	<.001
Sup 3L – Sup 1L	1.132	.103	Inf	10.956	<.001
No 3L – No 2L	-.188	.084	Inf	-2.244	.218
No 2L – No 1L	1.531	.136	Inf	11.278	<.001
No 3L – No 1L	1.344	.143	Inf	9.356	<.001
Sup 3L – No 3L	-.211	.133	Inf	-1.584	.609
Sup 2L – No 2L	-.849	.121	Inf	-6.993	<.001
Sup 1L – No 1L	-1.095	.143	Inf	-7.621	<.001

Note. Sup = Support, No = No Support, 3L = Third last Leaf; 2L = Second last Leaf; 1L = Last Leaf. se = Standard Error, df = Degrees of Freedom.

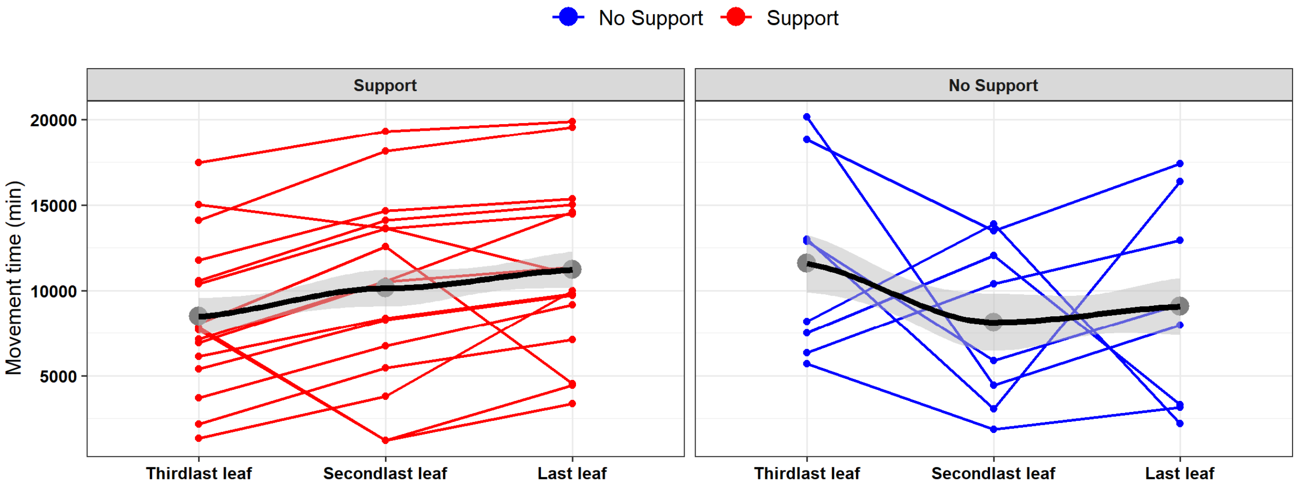

Figure S3. Graphical representation for the variation of the movement time across the last three leaves developed (in columns) per each Experimental Condition (in facets, 'Support' and 'No support'), controlling for individual plants (by line). Per each condition, the black dots represent the mean movement time for each leaf, with the black continuous line representing the mean smoothed variation across leaves. Plants for the 'Support' condition are represented with red-solid lines, while those for the 'No Support' condition have blue-solid lines.

Table S5A. Mean, standard deviation, and range (min, max) concerning movement time (min) of leaves, controlling for Experimental Condition ('Support,' 'No Support') and Leaf ('Third last,' 'Second last,' 'Last').

	Mean (SD)	Min	Max
**Condition: Support**			
- Third last leaf	8481.7 (4528.2)	1359	17466
- Second last leaf	10132.5 (5501.9)	1218	19299
- Last leaf	11208 (5016.2)	3369	19902
**Condition: No Support**			
- Third last leaf	11578.5 (5601.5)	5706	20169
- Second last leaf	8135.2 (4872.1) 9063	1860	13884
- Last leaf	(6029.5)	2193	17412

Note. One day (24h) = 1440 mins, and ten days (240h) = 14400 mins.

Table S5B. Results from the lmer fitted models (Type III Wald chi-square tests) investigating the interaction between Experimental Condition ('Support,' 'No Support') and Leaf ('Third last,' 'Second last,' 'Last') for the movement time. The plant was set as a random intercept of the model.

	**$\chi 2$**	**df**	**Pr(>$\chi 2$)**	**R^2**
**Leaf movement time ~**				
(Intercept)	24.37	1	<.001***	
Leaf	4.054	2	.131	
Condition	0.910	1	.168	
Leaf *Condition	7.584	1	.022*	
- Marginal R^2				.056
- Conditional R^2				.561

Note. χ^2 = chi-squared test; R^2= Coefficient of determination; df: degree of freedom; ° = $p < .100$; * = $p < .050$; ** = $p < .010$; *** = $p < .001$.
